# Fundamental Flaws of Hormesis for Public Health Decisions

**DOI:** 10.1289/ehp.7811

**Published:** 2005-06-15

**Authors:** Kristina A. Thayer, Ronald Melnick, Kathy Burns, Devra Davis, James Huff

**Affiliations:** 1 National Institute of Environmental Health Sciences, National Institutes of Health, Department of Health and Human Services, Research Triangle Park, North Carolina, USA; 2 Sciencecorps.org, Lexington, Massachusetts, USA; 3 H. John Heinz III School of Public Policy & Management, Carnegie Mellon University, Pittsburgh, Pennsylvania, USA

**Keywords:** biphasic dose response, hormesis, individual susceptibility, low-dose exposures, nonmonotonic dose response, nonlinear dose response, public health, regulation, risk assessment

## Abstract

Hormesis (defined operationally as low-dose stimulation, high-dose inhibition) is often used to promote the notion that while high-level exposures to toxic chemicals could be detrimental to human health, low-level exposures would be beneficial. Some proponents claim hormesis is an adaptive, generalizable phenomenon and argue that the default assumption for risk assessments should be that toxic chemicals induce stimulatory (i.e., “beneficial”) effects at low exposures. In many cases, nonmonotonic dose–response curves are called hormetic responses even in the absence of any mechanistic characterization of that response. Use of the term “hormesis,” with its associated descriptors, distracts from the broader and more important questions regarding the frequency and interpretation of nonmonotonic dose responses in biological systems. A better understanding of the biological basis and consequences of nonmonotonic dose–response curves is warranted for evaluating human health risks. The assumption that hormesis is generally adaptive is an oversimplification of complex biological processes. Even if certain low-dose effects were sometimes considered beneficial, this should not influence regulatory decisions to allow increased environmental exposures to toxic and carcinogenic agents, given factors such as interindividual differences in susceptibility and multiplicity in exposures. In this commentary we evaluate the hormesis hypothesis and potential adverse consequences of incorporating low-dose beneficial effects into public health decisions.

The concept of hormesis has received considerable attention over the past several years (Kaiser 2003a, 2003b). A recent literature search in the PubMed database on the term “hormesis” yielded 215 papers published between 2000 and 2004 compared to 116 published in 1999 and earlier (PubMed 2005). In several commentaries and reviews, hormesis—defined as low-dose stimulation, high-dose inhibition—has been used to promote the notion that low-level exposures to known toxic chemicals could be “beneficial” to human health (Calabrese and Baldwin 2003c; Renner 2004). For example, it has been proposed that

if low-dose stimulatory responses were assumed to be beneficial, the decision maker could view hormesis as adding potential benefit to society and could estimate an optimized population-based exposure standard. (Calabrese and Baldwin 2003a, p. 188)

Some proponents of this view claim hormesis is an adaptive, broadly generalizable phenomenon and argue that in the absence of contradictory information, the default assumption for risk assessments should be that at low exposures, toxic chemicals induce stimulatory effects (Calabrese and Baldwin 2003a). We argue that many examples used to support the widespread frequency of hormesis are better described by the more general term “nonmonotonic” dose responses. Nonmonotonic is used to describe dose–response relationships in which the direction of a response changes with increasing or decreasing dose. Use of the term hormesis, with the associated descriptors of low-dose stimulation and high-dose inhibition, can only be justified if there is an understanding of the biological processes underpinning that specific dose response. We agree that there is a need to address nonmonotonic dose–response relationships in the risk assessment process. However, even if certain low-dose effects were sometimes determined to be beneficial, this finding should not be used to influence regulatory decisions to increase environmental exposures to toxic agents, given factors such as variability in individual susceptibility, variability in individual exposures, and the public’s regular exposure to complex mixtures. Our commentary focuses on the evaluation of the hormesis hypothesis and consequences of incorporating low-dose beneficial effects into public health decisions, with special emphasis on the following issues:

- *The concept of hormesis is based largely on empirical observations and does not adequately consider underlying mechanism(s) of action.* Without an understanding of the mechanisms underlying a hormetic response, it is not appropriate to conclude that hormesis is a uniformly adaptive phenomenon.
- *Stimulatory responses are not always beneficial, and some may be harmful.* There is no scientific support for the assumption that stimulatory responses such as increased growth, enzyme activity, hormone concentration, and cell proliferation are beneficial.
- *Health decisions based on beneficial effects must address all the induced effects by that agent.* Examples cited to support the incorporation of low-dose beneficial effects into exposure standards ignore other adverse effects that are induced by different mechanisms and that occur at similar or lower dose levels.
- *Health decisions based on beneficial effects must address interindividual differences in exposure and susceptibility, including genetic, life-stage, and health status factors.* Susceptibilities and exposure levels vary among people over the course of a lifetime. In many cases timing of exposure can be more important than dose in determining health outcomes. Fundamental physiological differences stemming from genetic heterogeneity and differences in health status will also influence susceptibility.
- *Health decisions based on beneficial effects must address the fact that other environmental and workplace exposures may alter the low-dose response of a single agent.* Exposures in the real world do not occur to single substances but to mixtures of toxicants that can interact with each other or affect different steps of multistage disease processes. The mix of chemicals that individuals are exposed to varies depending on the nature of their work, indoor home environment, drinking water supply, food sources, school environment, and where they socialize, in addition to lifestyle choices such as diet, hobbies, hygiene practices, and other factors such as the use of prescription and over-the-counter drugs. Moreover, many of these compounds can affect the same target tissues by either similar or different mechanisms of action.

## The Concept of Hormesis As an Adaptive Response Does Not Adequately Consider Underlying Mechanisms of Action

**Figure 1:**
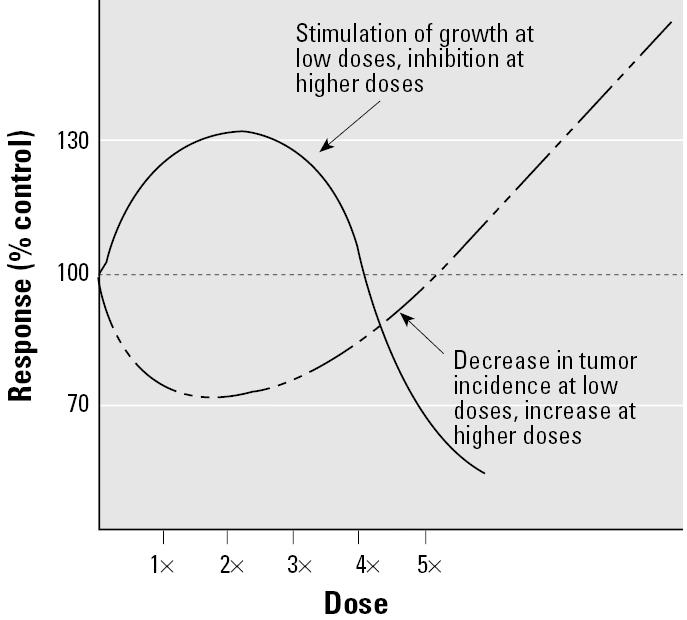
Nonmonotonic dose response for growth or cancer incidence.

As already stated, hormesis is generally described as low-dose stimulation and high-dose inhibition, producing a nonmonotonic dose response. This may be visualized in the situation in which low-dose exposure to an agent stimulates growth and high-dose exposure inhibits growth (Figure 1, solid line) (Renner 2004). U- or J-shaped dose responses (Figure 1, dashed line) can also be considered hormetic. A more recent definition of hormesis by Calabrese and co-workers considers the phenomenon to be an “adaptive” and frequently observed response resulting from exposure to a perturbing agent (Calabrese and Baldwin 2002b). Many of the recent publications on hormesis and its application to risk assessment are coauthored by Calabrese or reference his work. Thus, the works of Calabrese and his colleagues at the University of Massachusetts-Amherst are cited frequently in this article. These authors assert that

the hormetic phenomenon response is a common, evolutionary-based strategy to carefully regulate resource allocation in a definable range within the context of the re-establishment and maintenance of homeostasis. (Calabrese and Baldwin 2002a, p. 333)

In general this definition has positive (i.e., beneficial) connotations in that it implies that by adapting or coping with a stressor one will not suffer ill consequences. Yet, the evaluation criteria used to conclude that hormesis is a widespread, adaptive phenomenon are based on empirical observations of dose–response relationships with no regard for underlying mechanism(s) (Calabrese and Baldwin 2001, 2003).

Calabrese and Baldwin attempted to evaluate the frequency of hormesis by first reviewing studies published in three journals [*Environmental Pollution* (1970–1998), *Bulletin of Environmental Contamination and Toxicology* (1966–1998), and *Life Sciences* (1962–1998)] that they believed to represent a broad range of experimental models (Calabrese and Baldwin 2001). Epidemiologic and field studies were excluded from this analysis, as were non-English language articles. The authors evaluated 668 dose–response relationships from 195 published articles for evidence of hormesis that met the following inclusion criteria: *a*) presence of a concurrent control; *b*) capacity to achieve responses greater than (or less than) the control response; *c*) at least stwo doses below the no observed adverse effect level (NOAEL); and *d*) at least one dose showing *a priori* criteria-based inhibition (Calabrese and Baldwin 2001). The NOAEL was defined as either *a*) the highest dose with a response not statistically different with respect to adverse response from the control or *b*) the highest dose with a response ≥90% of control for inverted U-shaped dose–response relationship or as the highest dose with a response ≤110% of control for U- or J-shaped dose–response relationships. *A priori* criteria-based inhibition occurs when *a*) the response for at least one dose higher than the NOAEL is statistically different from controls, *b*) the response for at least one dose higher than the NOAEL shows a change of twice the value of the SD or SEM compared to the control group (for studies where only data distribution is reported); and *c*) the response for at least two doses higher than the NOAEL is < 90% of the control for inverted U-shaped dose–response relationships or > 110% of the control for U- or J-shaped dose–response relationships.

A dose–response relationship was considered hormetic if *a*) at least one dose at or below the NOAEL was statistically increased (for inverted U-shaped dose–response relationships) or decreased (for J- or U-shaped dose–response relationships); *b*) at least three doses at or below the study NOAEL had responses ≥110% of the control (for inverted U-shaped dose–response relationships) or ≤90% of the control (for J- or U-shaped dose–response relationships); or *c*) for studies in which only data distribution is reported, variability in response (2 times the SD or SEM) did not overlap with variability in the control group.

**Figure 2:**
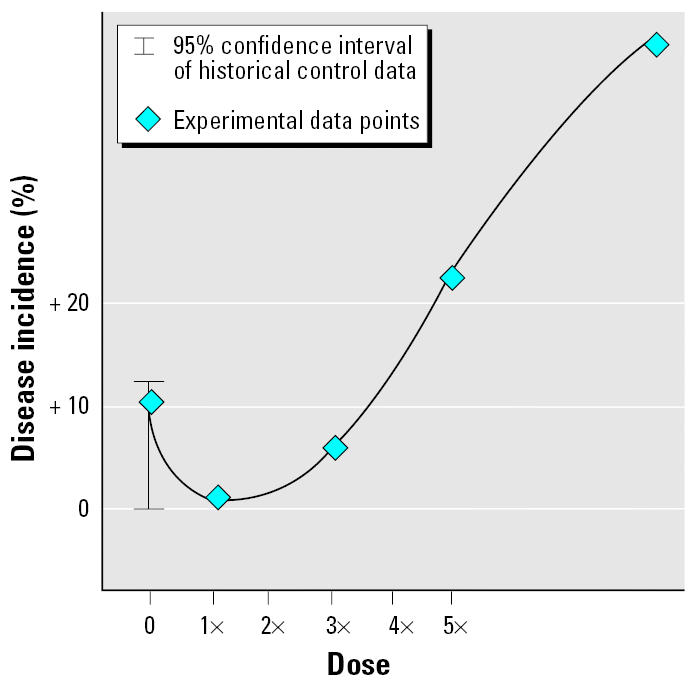
An apparent hormetic response may reflect data variability. Some responses may appear to be hormetic but actually be an artifact of the experimental and analytical methodology because of data variability (shown here), small group size, large number of end points analyzed, unequal evaluations in all dose groups, effects of the agent on body weight and survival, and the underlying mechanism of the nonmonotonic dose response. Criteria for listing a response as hormesis must address all these potential confounding factors.

Using a study NOAEL to determine whether there are stimulatory effects at or below that dose is problematic because the determination of a NOAEL whether by selecting one of the actual doses in a study (non-significant change from control) or by modeling the dose–response data is influenced by the variability in the experimental data, sample size, the statistical power of the study, the end point being evaluated, the duration and route of exposure, and so forth. Because of variability in the control response (reflected in historical control data), a difference in response between the current control group and the dose groups below the presumed NOAEL may give the false appearance of a hormetic response. Thus, in some cases an apparent hormetic response may simply reflect data variability (Figure 2) rather than low-dose stimulation and high-dose inhibition.

The evaluation criteria used by Calabrese and Baldwin to determine whether a dose response is hormetic do not require statistically significant changes from control. Many of the dose responses classified as hormetic were identified based on the criteria that at least three doses at or below a study NOAEL differ by ≥10% of the relative control response (Calabrese and Baldwin 2001). For example, a change in incidence from 20 of 100 (20%) to 18 of 100 (18%) would be interpreted as a 10% change from control response [(20–18) of 20] and not a 2% change (20–18) in response. This approach can lead to a large change relative to the control with only a one-count change in response (e.g., the difference between 3 of 20 and 2 of 20 would amount to a 33% change). In this manner even small changes in incidence that reflect data variability would be interpreted incorrectly as evidence to support the widespread occurrence of hormesis.

**Figure 3:**
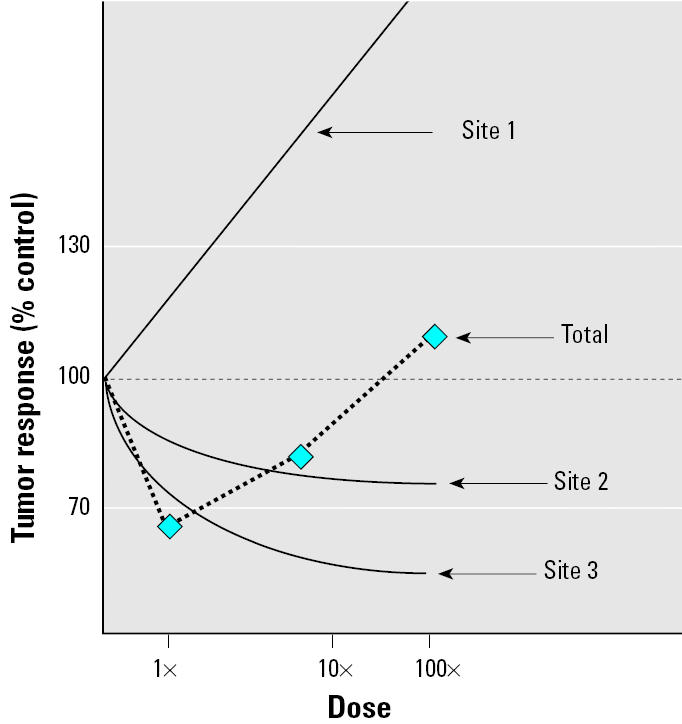
Three nonhormetic responses do not equal hormesis. The increase in tumor response at site 1 and the decreases at sites 2 and 3 are monotonic and therefore nonhormetic. Although the total tumor response appears to be nonmonotonic, this is not hormesis.

In some cases the apparent hormetic response reported in animal studies may be largely an artifact of the evaluation methodologies. For example, 2,3,7,8-tetrachloro-*p*-dibenzodioxin (TCDD) has been frequently cited as an environmental carcinogen that produces low-dose beneficial effects (Calabrese and Baldwin 2003c;Kaiser 2003). In the carcinogenicity study of TCDD (Kociba et al. 1978), the incidence of tumors of the liver, lung, tongue, and nasal turbinates were increased, and the incidence of tumors of the pituitary, uterus, mammary glands, pancreas, and adrenal gland were decreased. In no case was an individual tumor response nonmonotonic; however, by calculating the total number of tumors, Calabrese presents the overall tumor response as hormetic (Kaiser 2003b). We argue that this should not be considered hormesis because none of the specific tumor responses contributing to the shape of the total tumor dose response can be considered hormetic or nonmonotonic. A simplified version of this scenario is presented in Figure 3.

There are additional issues regarding the interpretation of the dose–response data for total tumor incidence in the TCDD study. In that study (Kociba et al. 1978), mortality was increased in the high-dose group, and body weights were decreased relative to that of controls. Because adjustments were not made for early mortality, estimations of total tumor rates relative to controls are not reliable. In addition, it is well known that lower body weight is associated with reduced tumor incidence at several sites (Rao et al. 1987). Further, histologic examinations in the low-dose and mid-dose groups were not as extensive as those performed for the control and high-dose groups. Thus, the apparent hormetic response is not based on reliable data.

## Stimulatory Responses Are Not Always Beneficial, and Some May Be Harmful

Although Calabrese and Baldwin (2002b) state that the adaptive response should not be interpreted *a priori* as being either beneficial or harmful, in other publications they claim that dose stimulatory responses are generally beneficial. For example:

Acceptance of hormesis will be difficult, therefore, because agencies will need to accept the possibility (*actually, the likelihood*) that toxic substances, even the most highly toxic (e.g., cadmium, lead, mercury, dioxin, PCBs, etc.) can cause beneficial effects at low doses [emphasis added]. (Calabrese and Baldwin 2003a, p. 191)

In any case, adaptive responses may be beneficial or harmful depending on the life stage or circumstances under which they occur. For example, natural hormones are responsible for maintaining homeostasis and controlling normal development; hence, exposure to agents that interfere with homeostatic control processes, especially those that stimulate growth at inappropriate or vulnerable times, can lead to abnormal development.

The concept of hormesis is based on experimental observations, but the assumption that stimulatory effects are always or usually beneficial is unproven. Many low-dose stimulatory responses with equally likely adverse consequences include increased cell replication, DNA synthesis; blood pressure, heart rate, interleukin-2 release, prolactin release, testosterone concentration, luteinizing hormone concentration, and dopamine outflow (Calabrese and Baldwin 2003b).

The concept of radiation hormesis is based on the hypothesis that low-dose ionizing radiation induces adaptive responses that enhance the repair of DNA damage from endogenous and exogenous sources and stimulate cell removal (Pollycove and Feinendegen 2003). However, this hypothesis needs to be tested. In addition, it is necessary to recognize that adaptive stress responses such as enhanced cell death may be beneficial or harmful depending on the circumstance of the response, and interpretations of hormetic effects of radiation exposure may be influenced by experimental designs. For example, the report of a negative correlation between domestic radon exposure and lung cancer mortality (Cohen 1995) was likely due to failure to account for confounding by cigarette smoking (Puskin 2003). Moreover, two recent reports refute the credibility of “radiation hormesis” by concluding that low doses of radiation present a cancer risk [National Research Council (NRC) 2005; International Agency for Research on Cancer 2005]. Regarding the possibility of low dose beneficial effects, the NRC concluded that

the assumption that any stimulatory hormetic effects from low doses of ionizing radiation will have a significant health benefit to humans that exceeds potential detrimental effects from the radiation exposure is unwarranted. (NRC 2005, p. 585)

Studies reviewed in support of the radiation hormesis hypothesis were “found either to be based on ecologic studies or to cite findings not representative of the overall body of data” (NRC 2005, p 19).

There are other clear examples where a stimulatory effect would not be considered beneficial. For example, agents that induce cytochrome P450 activities to enhance the rate of elimination of xenobiotics will also increase the mutagenic potential of chemicals that are activated to DNA-reactive intermediates by these enzymes. Glutathione *S*-transferase (GST) is usually considered to be a detoxifying enzyme. However, GST-mediated glutathione conjugation of trichloroethylene and other haloalkenes produces mutagenic intermediates. Thus, in some cases increased GST activity may be beneficial while in other cases it may be harmful. Polymorphisms in genes coding for metabolizing enzymes contribute to interindividual variability discussed below and may vary by more than 50-fold in humans (Guengerich et al. 1991).

*In utero* exposure to low and high doses of the synthetic estrogen diethylstilbestrol (DES) has opposite effects on uterine response to hormonal stimulation in adulthood (Alworth et al. 2002). Although at low doses the effect is stimulatory (increased uterine size) and therefore fits within the original definition of hormesis, this effect is not beneficial. In fact, a chemically induced positive uterotropic response is used as a screen for estrogenicity and raises concern about the toxicity of the agent [U.S. Environmental Protection Agency (EPA) 1998].

## Health Decisions Based on Beneficial Effects Must Address All Induced Effects

**Figure 4:**
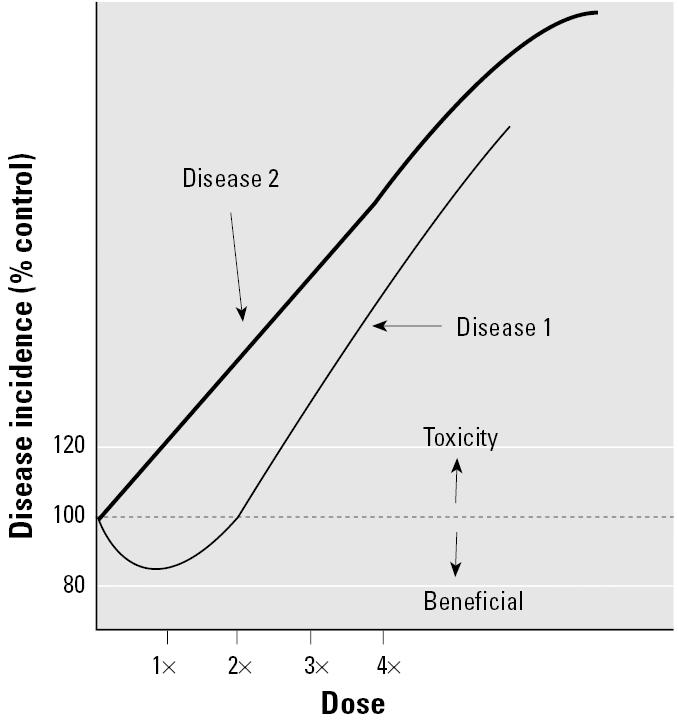
An agent induces multiple effects. An apparent beneficial hormetic dose for disease 1 (at dose 1×) increases disease incidence for disease 2. For example, an agent may induce liver tumors at the same low dose that is associated with a decrease in pituitary tumors.

The idea of focusing primarily on purported beneficial hormetic responses when making decisions for exposure standards is greatly weakened when all the toxicologic and epidemiologic evidence for a given compound or agent is considered. A major concern is that an agent may produce an apparent low-dose beneficial response for one effect but also induce an adverse effect at that same dose in a different organ or another species (Figure 4). For example, cadmium has been touted as a model hormetic agent (Calabrese and Baldwin 2003c), partly because low experimental doses (1–10 μmol/kg) have been associated with-nonstatistically significant decreases in testicular tumors in rats (Waalkes et al. 1988). However, a significant increase in the incidence of prostatic neoplasias and an increase in the number of prostate tumors per animal were observed in this same study within the hormetic dose range (Waalkes et al. 1988, 1997). Notably, cadmium has been long recognized as being carcinogenic to humans, associated with prostate, lung, renal, and bladder cancers (National Toxicology Program 2002).

Moreover, three epidemiologic studies indicate that current exposures to cadmium in the general population are associated with adverse health outcomes (Matsuda et al. 2002; Satarug and Moore 2004; Schwartz et al. 2003). One of the studies reported that increasing levels of urinary cadmium are associated with impaired fasting glucose (pre-diabetes) and diabetes after adjusting for age, ethnicity, sex, and body mass index in a sample of more than 8,700 adults (Schwartz et al. 2003). These findings are consistent with animal data showing that cadmium causes damage to the pancreas and alters glucose regulation in laboratory animals (Han et al. 2003; Kanter et al. 2003; Merali and Singhal 1980). Cadmium and many other heavy metals are also fundamentally toxic to the kidneys, with chronic low-level exposure leading to tubular damage (Goyer 1991). This damage is associated with increased mortality (standardized mortality ratios) in areas such as Jinzu, Japan (Matsuda et al. 2002). Among individuals with limited kidney function and among many elderly people whose kidney function declines as they age, exposure to cadmium and other nephrotoxins, even at very low levels, can prove extremely dangerous. When all these findings are considered, it is improbable that allowing higher levels of cadmium in the environment would provide an overall health benefit for the general population.

Other purported hormetic agents such as radiation present the same concern. Noncancer health concerns include decreased birth weight (Hujoel et al. 2004) and cognitive impairment after prenatal radiation exposure (Hall et al. 2004; Otake and Schull 1998; Yamazaki and Schull 1990).

## Health Decisions Must Address Interindividual Differences in Exposure and Susceptibility

Regulating to achieve a purported beneficial response would require standards to be set at a specified level rather than below an exposure level. This would require that exposure levels in the general population be maintained within a narrow window which would be impossible. Even at a given environmental standard, differences in body mass can result in significant differences in exposure. For example, on a body-weight basis compared to adults, children breathe 3 times as much air, drink up to 7 times as much water, and ingest 3 times as much dust and soil because they put their hands in their mouths frequently (U.S. EPA 1997, 2002). The National Academy of Sciences Committee on Pesticides emphasized the importance of exposure in accounting for the differences in pesticide-related health risks between children and adults (National Academy of Sciences 1993).

**Figure 5:**
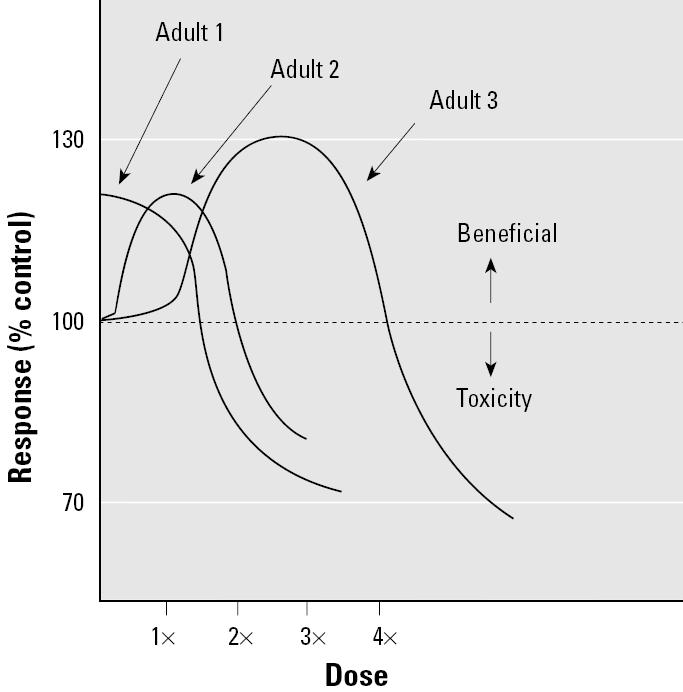
Interindividual variability. An apparent maximal beneficial hormetic dose for adult 3 (at dose 3×) is toxic to adult 1 and adult 2. Because of genetic differences and extrinsic factors, people may respond differently to environmental toxicants. In this figure, adult 1 receives no benefit with any exposure to the agent and the dose response for adult 2 is maximal at a lower dose than that for adult 3.

**Figure 6:**
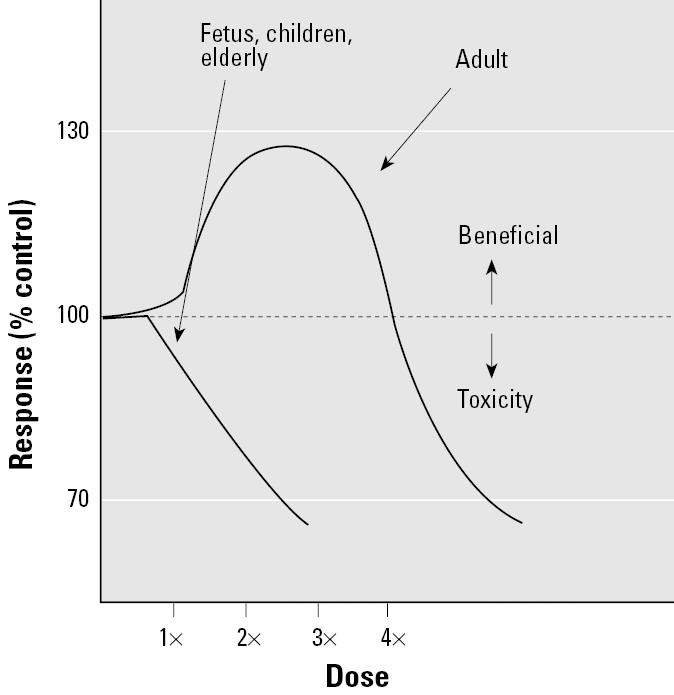
Life stage differences in susceptibility. If the fetus, children, elderly, or other groups do not experience a beneficial hormetic response, health decisions based on hormesis will result in higher risks for these populations.

Susceptibilities vary among individuals and over the course of a lifetime, making it difficult to identify a beneficial hormetic exposure at the population level. Based on numerous intrinsic and extrinsic factors that affect interindividual susceptibility to toxic agents, a dose that may appear to be beneficial for one subgroup (e.g., healthy young males) may produce adverse health effects in other subgroups (e.g., children, the elderly, immune-compromised individuals, or workers exposed to other toxic agents; Figures 5 and 6).

Consider ethanol, which is cited as a classic hormetic agent because low or moderate drinking is associated with beneficial outcomes including reduced overall mortality and reduced risk of coronary heart disease (CHD) and stroke, whereas high consumption is associated with other types of heart diseases, neurological disorders, cancer, liver cirrhosis, and traffic accidents (Agarwal 2002). But low to moderate drinking in pregnant women (defined as 1.2–2.2 drinks per day) is discouraged because even small amounts of alcohol during pregnancy (0.5 drinks per day) have been associated with adverse behavioral outcomes in children, including aggressive behavior (Sood et al. 2001). Because no evidence exists for thresholds of risk-free drinking during pregnancy, the American Academy of Pediatrics and the American College of Obstetrics and Gynecologists recommend abstinence for preconceptional and pregnant women (Sokol et al. 2003). Health decisions based on a limited characterization of variability in hormetic responses among exposed individuals may result in excessive health risks for susceptible subpopulations who do not experience the same dose-related effects.

A recent analysis of experimental animal studies for four types of ionizing radiation (Cs-137 gamma rays, X rays, neutrons, and internal βrays resulting from the injection of tritiated water) estimated a 3.5- to 5.3-fold increase in carcinogenic sensitivity per dose when exposure occurred in the fetal to birth–weaning period relative to comparable doses in adults (Hattis et al. 2004). In addition to lifestage differences in susceptibility to radiation-induced cancer, tumor response to radiation in adult animals varies depending on strain (Broerse et al. 1986), hormone status (i.e., estrogen levels; Bartstra et al. 2000), and whether the dose of radiation is a single or fractionated exposure (Maisin et al. 1988). There are many reasons that fetuses, infants, and children are more sensitive to chemicals than are adults. These range from the well-known susceptibilities of developing organ systems, such as the nervous system to neurotoxins including lead (Agency for Toxic Substances and Disease Registry 1999) and mercury (NRC 2000), as well as to age-related differences in metabolism and elimination (Ginsberg et al. 2002).

In addition to differences in exposure, age and genetic variabilities are relevant to consideration of the toxicity of organophosophate (OP) pesticides that are present in food and pet treatments. The enzyme paraoxonase (PON) metabolizes toxic breakdown products of OPs. People with higher than average PON levels due to genetic polymorphisms metabolize OPs more quickly (Hulla et al. 1999). Infants are especially vulnerable to OPs because adult levels of PON are not produced until approximately 2 years of age (Chen et al. 2003; Ecobichon and Stephens 1973). Other exposures such as alcohol, cigarette smoke, and certain medications also affect the level of PON-1 activity (Gouedard et al. 2003; Wang et al. 2004). Similarly, OP detoxification by malaoxonase differs between adults and children and varies at least 7-fold among adults (Sams and Mason 1999). Health decisions that do not adequately account for human variability will not sufficiently protect vulnerable segments of the general population.

## Health Decisions Must Address Other Environmental and Workplace Exposures

**Figure 7:**
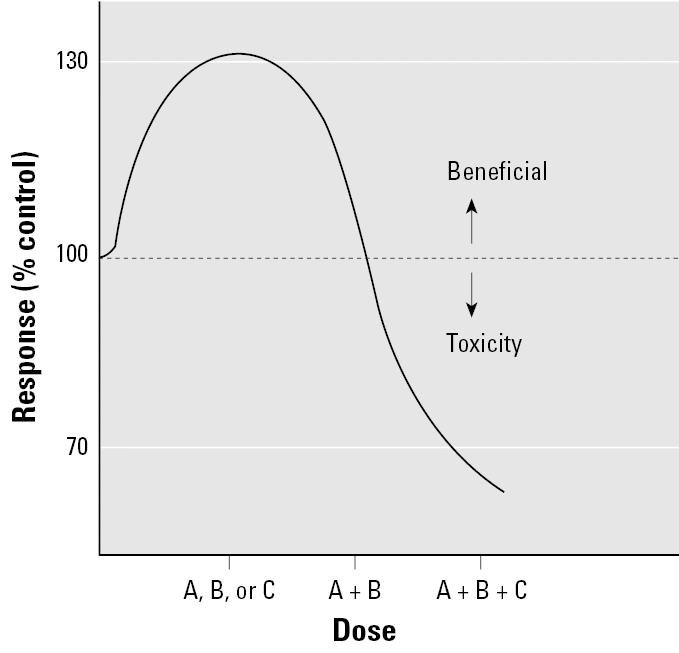
Exposure to mixtures. If agents A, B, and C act by similar mechanisms (e.g., activate the same receptor), then exposure to apparent beneficial hormetic doses of each of these together is toxic. In this example low-dose exposure to two agents may not produce a beneficial effect, but low-dose exposure to three agents is toxic. Because we are all exposed to different mixtures of toxic agents, beneficial health effects in the real world cannot be assumed based on responses of individual agents.

Advocates of incorporating beneficial hormetic responses into risk assessment fail to recognize that people are exposed to hundreds of compounds each day, and these vary depending on our environmental and occupational exposures. According to Calabrese, maximal low-dose hormetic response stimulation for a given chemical occurs on average at a dose 5-fold below the NOAEL (Renner 2004). Thus, it follows that simultaneous exposure to other compounds that elicit similar toxic responses would be enough to move an individual from the low-dose supposed beneficial range to the range where adverse effects are expected (Figure 7). For example, a decision based on an apparent low-dose beneficial effect for TCDD would increase health risks because the general population is exposed to numerous dioxin-like compounds that also induce disease through activation of the aryl hydrocarbon receptor. Given that residues of hundreds of chemicals have been measured in humans (3M 2002; Centers for Disease Control and Prevention 2003; Environmental Working Group 2003; Olsen et al. 2002; Schecter et al. 2003), with many of them affecting the same tissues and fluctuating in concentration over the course of a lifetime, titrating exposure to achieve a relatively narrow beneficial hormetic range is untenable and clearly a poor public health policy.

## Conclusions

Only after careful consideration of the biological underpinnings of a truly beneficial response can an exposure be considered for the general population, such as the addition of folic acid to cereals. If a toxic or hazardous pollutant were found to have truly beneficial effects at low dose, then that agent should be tested clinically, go through the U.S. Food and Drug Administration (FDA) approval process, and be regulated as a pharmaceutical for those who might benefit from its use. Certainly, the general population should not be exposed to chemotherapeutic agents that benefit cancer patients. For pharmaceuticals, it is understood that there are trade offs between benefits and risks. For example, although aspirin is a generally well-tolerated pain reliever and is increasingly advocated as a preventative tool for heart attacks and colorectal cancer (Vainio and Miller 2003; Werner et al. 2004), it is also linked to increased risk of gastrointestinal bleeding, cerebral hemorrhage (Werner et al. 2004), and asthma attacks (Jenkins et al. 2004). In addition, aspirin is not recommended for children or teenagers who have or are recovering from chicken pox or flulike symptoms because it can cause debilitating and sometimes lethal Reyes syndrome (U.S. FDA 2003). Individual risks to pharmaceutical agents can be controlled with proper usage; however, increased exposure to environmental toxins presents additional involuntary risks for the general population. Under the latter condition, exposure is inadequately controlled, and there is no mechanism to correct for individual circumstances (e.g., medical condition or age) that may result in harm.

Although hormetic effects may occur in some instances, it is indeed rare that exposures to toxic, mutagenic, teratogenic, and carcinogenic chemicals, even at low exposure levels, would be risk free and provide health benefits for the general public. Portraying chemicals with numerous adverse effects as having benefits while ignoring their hazards is irresponsible and does not provide full and objective disclosure. In the 1950s doctors prescribed DES to pregnant women to prevent miscarriage and premature births and to produce “bigger and stronger babies” even though DES had been shown to cause damage to reproductive tissues in animals (Dinusson et al. 1948; Dunn and Green 1963; Takasugi and Bern 1964). Human use of DES was banned in the United States in 1971 after the discovery of high rates of rare, clear-cell adenocarcinomas of the vagina and cervix in DES-exposed daughters (Herbst 1981), and later studies showed elevated breast cancer risk in women who took DES during pregnancy (Titus-Ernstoff et al. 2001). Certainly, health policy decisions should be based on scientific evidence and not on speculation of health benefits in order for the general population to avoid repeating the mistakes of the past similar to that of the DES tragedy.

The claims and projections of health benefits from exposures to environmental toxicants and carcinogens are based on untested assumptions and disregard numerous well-established scientific principles that underpin a public health–protective approach to regulating exposure to toxic substances. If hormesis were used in the decision-making process to allow higher exposures to toxic and carcinogenic agents, this would substantially increase health risks for many, if not most, segments of the general population.
